# The Impact of Public Health Interventions on the Frequency and Duration of Hospitalisations Among Seniors in Poland—An Analysis Covering the Years 2017–2018

**DOI:** 10.3390/jcm15010040

**Published:** 2025-12-20

**Authors:** Katarzyna Kwiatkowska, Monika Pajewska, Olga Partyka, Aleksandra Czerw, Dorota Charkiewicz, Łukasz Strzępek, Mateusz Curyło, Magdalena Zawadzka, Monika Urbaniak, Katarzyna Sygit, Sławomir Porada, Izabela Gąska, Elżbieta Kaczmar, Jarosław Drobnik, Piotr Pobrotyn, Dorota Waśko-Czopnik, Tomasz Sowiński, Urszula Grata-Borkowska, Katarzyna Tejza, Ewa Bandurska, Weronika Ciećko, Elżbieta Grochans, Anna M. Cybulska, Daria Schneider-Matyka, Monika Borzuchowska, Karolina Kamecka, Remigiusz Kozlowski

**Affiliations:** 1 Department of Economic and System Analyses, National Institute of Public Health NIH—National Research Institute, 00-791 Warsaw, Poland; 2 Department of Health Economics and Insurance, Center for the Humanities and Social Sciences of Medicine, Medical University of Warsaw, 00-581 Warsaw, Poland; 3 Department of Surgery, Andrzej Frycz Modrzewski Cracow University, 30-705 Krakow, Poland; 4 Clinical Department of General and Oncological Surgery, Saint Raphael Hospital, 30-693 Krakow, Poland; 5 Institute of Health Sciences, University of the National Education Commission, 30-084 Krakow, Poland; 6 Psychotherapy Day-Care Unit, Hospital of the Ministry of Interior and Administration in Krakow, 30-053 Krakow, Poland; 7 Department of Education, Prevention and Health Promotion, Military Institute of Hygiene and Epidemiology, 01-163 Warsaw, Poland; 8 Department of Epidemiology and Public Health, Medical University of Lodz, 90-131 Lodz, Poland; 9 Department of Medical and Pharmaceutical Law, Faculty of Medicine, University of Medical Sciences, 61-701 Poznan, Poland; 10 Faculty of Medicine and Health Sciences, University of Kalisz, 62-800 Kalisz, Poland; 11 Faculty of Health Sciences and Psychology, Collegium Medicum, University of Rzeszów, 35-310 Rzeszow, Poland; 12 Medical Institute, Jan Grodek State University in Sanok, 38-500 Sanok, Poland; 13 Department of Family Medicine, Faculty of Medicine, Wroclaw Medical University, 51-141 Wroclaw, Poland; 14 Citodent Dental Center, Furtak-Pobrotyn & Company Limited Partnership, 05-220 Olawa, Poland; 15 Department of Gastroenterology, Hepatology with Inflammatory Bowel Disease Subunit, Provincial Specialist Hospital J. Gromkowskiego, 51-149 Wroclaw, Poland; 16 Department of Non-Surgical Clinical Sciences, Faculty of Medicine, Wrocław University of Science and Technology, 50-370 Wrocław, Poland; 17 Endocare Medical Center, Simple Joint-Stock Company (S.J.S.C.), 50-558 Wroclaw, Poland; 18 Center for Competence Development, Integrated Care and e-Health, Medical University of Gdansk, 80-204 Gdansk, Poland; 19 Department of Nursing, Faculty of Health Sciences, Pomeranian Medical University in Szczecin, 71-210 Szczecin, Poland; 20 Department of Management and Logistics in Healthcare, Medical University of Lodz, 90-131 Lodz, Poland

**Keywords:** older adults, healthcare costs, public health

## Abstract

**Background/Objectives**: Public health programmes for older adults aim to reduce hospital admissions and improve health outcomes. However, the effects of these programmes on the length of hospital stays for seniors remain unclear. This study aimed to examine the link between the number and type of public health initiatives implemented between 2017 and 2018, and the number of hospitalisations, as well as the duration of hospital stays in 2019 and 2020, among seniors with heart, digestive, and musculoskeletal diseases. **Methods**: A correlation analysis was conducted to explore the relationship between the number of public health programmes and activities, and the number and length of hospitalisations among older adults. Statistical significance was set at *p* < 0.05 and *p* < 0.01. **Results**: The analysis revealed positive correlations between the number of completed public health tasks and the length of hospital stay across the three disease groups. For heart disease, hospital stay length was correlated with the total number of programmes (r = 0.501, *p* < 0.05) and those specifically supporting medical services (r = 0.574, *p* < 0.05). In cases of digestive diseases, correlations were observed with the overall number of programmes (r = 0.623, *p* < 0.01), as well as programmes in the “general” category and ones supporting medical services (r = 0.544–0.601, *p* < 0.05). Regarding musculoskeletal diseases, the strongest correlation occurred with programmes that support medical services (r = 0.700, *p* < 0.01). **Conclusions**: Our results indicate that increased public health interventions may be associated with longer hospital stays among seniors, likely reflecting the emergence of more complex health needs and increased diagnostic intensity. At the same time, analysis based on ecological data does not allow for the establishment of causal relationships, emphasizing the need for further, more advanced research that controls for confounding factors.

## 1. Introduction

According to the World Health Organization, by 2030, one in six people worldwide will be over 60. By 2050, the global population aged 60+ will double to 2.1 billion, and the count of individuals aged 80+ will reach 426 million [1]. In OECD countries, the proportion of older individuals (aged 65+) is notably higher among highly developed nations, while it is significantly lower in developing countries [2].

### 1.1. Problem Statement

According to data in 2024, people aged 60 and over accounted for 26.6% of the entire Polish population [3]. OECD data indicates that among Poles aged 65+, as many as ~50% of men and ~60% of women have multiple chronic diseases [4]. In 2023, among hospitalized patients in Poland, people aged 60 and over accounted for 55.6% of the value of reimbursement for hospital treatment, which demonstrates the significant burden of this population segment on the hospital system [5]. A study by Pobrotyn et al., which examined over 300,000 hospitalizations, showed that the average length of hospital stay for patients ≥ 65 years was approximately 4.7 days, which is more than a day longer than for patients under 65 years of age. The average direct cost of hospitalization was also significantly higher [6].

In addition to the patient’s clinical condition, factors determining the length of hospitalization include demographic and social characteristics, as well as functional and organizational factors. Research indicates that the patient’s functional dependence in basic life activities, a higher number of comorbidities, polypharmacy, and cognitive impairment are significantly associated with a prolonged hospital stay [7]. Furthermore, socioeconomic status—lower education and less access to social support—is also associated with longer hospital stays, even after taking into account clinical determinants [8]. Organizational factors, such as the mode of hospital admission (e.g., urgent vs. elective) and delays in discharge planning, also influence the length of hospital stay, highlighting the complexity of determinants and the need for a multidimensional approach in epidemiological analyses and health policy.

Despite the growing number of public health programs targeted at older adults, there is still a lack of research clearly assessing the extent to which the diversity and intensity of these interventions influence the frequency and duration of hospitalizations among seniors. In particular, the mechanisms through which specific types of programs translate into a real reduction in the burden on the hospital system remain insufficiently understood. To date, no nationwide analyses have assessed how the scope and nature of public health interventions impact the frequency of hospitalizations for clearly defined categories of conditions among the senior population.

Filling this research gap is important as hospitalizations of older adults generate the highest costs in the healthcare system and increase the demand for already limited human resources. At the same time, frequent and prolonged hospital stays worsen seniors’ quality of life, contributing to a loss of independence and increased care needs. Given the dynamic aging of the population, understanding which public health measures actually reduce the number and severity of hospitalizations is crucial to maintaining the efficiency and sustainability of the healthcare system.

It is worth emphasizing that the duration of hospitalizations is as important as their number. Long hospital stays pose a significant burden for seniors, as they increase the risk of hospital-acquired infections and contribute to a decline in functional capacity and loss of independence. Hospitalization can also lead to mental deterioration, social isolation, and difficulty returning to pre-illness functioning [9]. As mentioned above, one of the key characteristics of geriatric patients is multimorbidity, which significantly contributes to prolonged hospital stays. Managing chronic diseases is, in turn, a crucial element of public health interventions aimed at seniors [10]. Improving disease control can reduce the severity of disease and the need for long hospitalizations. For example, cardiovascular disease management programs in China have been shown to reduce the average length of hospital stay for patients with coronary heart disease after their implementation [11]. Therefore, in studies on the effectiveness of public health programmes aimed at older people, it is crucial to take into account not only the number of hospitalisations, but also the length of stay, which better reflects the actual health and systemic burden.

This quantitative ecological correlation study aims to assess the relationship between the implementation of public health programs and hospitalization rates for people aged 65+ using Pearson correlation analysis. The primary goal of the study was to assess how the number and type of implemented public health programs influence the number of hospitalizations for specific disease groups. The goal is also to examine whether different forms of public health interventions are associated with changes in the duration of these hospitalizations, which has direct implications for seniors’ quality of life and the burden on the healthcare system.

The importance of this research objective stems directly from the knowledge gap described earlier: although hospitalizations for seniors represent a major financial and organizational burden, it remains unclear which public health interventions actually reduce their number and duration. The proposed study fills this gap by systematically assessing the impact of the number and type of public health programs on hospitalization rates, allowing us to determine which initiatives are most effective and where to direct resources in an aging society.

The study’s aim also fits into current scientific and policy debates regarding the role of prevention, coordinated care, and community-based services in improving the health of older adults. International analyses indicate that such interventions can reduce the need for hospitalization, and the OECD emphasizes the importance of shifting care from hospitals to the community [12]. Similar conclusions are drawn from studies of older people with chronic diseases, where public health services improved health and quality of life [13].

In recent years, there has been growing interest in the effectiveness of public health programs and community-based services for older adults, but the results often indicate that the effect of these interventions is mixed. For example, a systematic review of 58 programs targeting seniors found that comprehensive care centers and service coordination typically yielded better outcomes, while educational programs often provided limited benefits [14]. In contrast, studies of home-based interventions, such as nurse visits for people with multimorbidity, indicate potential but also reveal significant disparities in outcomes regarding quality of life, service utilization, and costs [15]. In this light, this study contributes to the current trend by attempting to verify whether intensification of public health interventions actually translates into improvements in hospitalization rates.

### 1.2. Significance of the Study

Over the past few decades, we have observed a rapid increase in the number of older people. In OECD countries, the percentage of people aged 65 and over increased from less than 9% in 1960 to 18.5% in 2023 [16]. In Poland, in 2024, nearly 10 million people were 60 years old and older, and by 2060 this population may increase to almost 12 million, constituting nearly 40% of the total population [3]. At the same time, the ageing of the population translates into a growing demand for long-term care and health services, which is associated with increasing public spending and pressure on human resources [16]. In this context, research on the impact of public health programs on hospitalizations of seniors becomes urgent as it can indicate which investments in prevention and care bring real savings, reduce the burden on hospitals and improve the quality of life of older people.

The study’s findings could be relevant to several stakeholder groups. The primary stakeholders of this study are health policymakers, who could use the findings to redesign public health programs to more effectively reduce hospitalizations among seniors. Hospital administrators may also be interested, as they could better plan resources and strengthen outpatient services or coordinated care. Local authorities could target investments in preventive care and community-based programs to regions where the greatest gap exists between seniors’ health needs and service availability. In the context of developing geriatric care, the study’s findings could be used to develop more tailored care models, focusing on the diseases and risk groups that contribute most to the burden of hospitalizations.

Although numerous reviews and empirical studies in recent years have demonstrated the potential of home care and community-based services, the empirical evidence remains patchy and population-specific. For example, a recent review of 42 international studies found wide variation in the use of home and community-based services, highlighting the need to individualize care based on the needs and characteristics of older adults [17]. In a 2024 review, which covered long-term care interventions, the authors point out that there is no convincing evidence that these programs systematically and permanently reduce the number of emergency department visits, hospital admissions, or other indicators of health care utilization [18].

The study results are important both for the academic community, broadening the understanding of the complexity of the senior care system, and for practitioners and decision-makers, who can use them to better design preventive programs and effectively manage hospital resources.

### 1.3. The Problem of Senior Hospitalization Length in the Polish Healthcare System

The Polish healthcare system is characterized by several structural and organizational features that may directly or indirectly influence the frequency and duration of hospitalization among seniors. First, the system is strongly oriented toward inpatient care, with a relatively high number of hospital beds compared to other OECD countries. This historically favors the use of hospitalization as the primary form of care delivery, rather than shifting some services to outpatient or community care. In 2025, Poland had over 6 hospital beds per 1000 inhabitants, significantly above the OECD average, which is combined with a low efficiency in the use of these resources and poor coordination between levels of care [16]. Another significant factor is the poor coordination between primary care, specialist care, and hospital care, which leads to many patients being admitted to hospitals due to insufficient outpatient support and late detection of health problems—particularly chronic conditions typical of the elderly population. Despite ongoing reforms, coordination of health services remains limited, and the percentage of same-day hospitalizations remains significantly lower than in countries with more centralized outpatient care [19].

Poland also experiences significant regional variations in access to healthcare, which can compromise outpatient care and indirectly lead to longer hospital stays for seniors. Analyses of the healthcare system indicate an uneven distribution of medical resources and limited access to outpatient and specialist services in some regions of the country, resulting in longer waiting times and greater burdens on hospital wards, especially due to older adults with chronic health conditions [4]. Additionally, data from the EuroHealth Observatory and OECD highlight that geographic and systemic inequalities in the availability of services—e.g., in diagnostics or primary care—can lead to delayed treatment and the need for more intensive hospital care, which in practice may extend the length of hospital stay for seniors with complex illnesses [19].

Shortages of medical personnel and limited long-term care resources are also significant problems, affecting the availability of health services and the quality of out-of-hospital care. Poland has one of the lowest ratios of long-term care workers per elderly among OECD countries, which can lead to more frequent and longer hospitalizations due to the lack of alternative care and rehabilitation options after discharge [4]. Research and expert reports also show that the Polish medical staff is relatively “old,” and its demographic structure will likely result in future shortages, which, given the aging population, poses a serious challenge to the healthcare system: In 2023, approximately 25% of doctors and 22% of nurses in Poland were at or near retirement age, further weakening human resources [20].

The OECD report highlights that Poland has some of the lowest spending on long-term care and a very limited supply of such services. Furthermore, approximately 40% of older people have unmet care needs, indicating significant gaps in Poland’s out-of-hospital care system [4]. Limited funding for preventive and outpatient services and long waits for specialist services contribute to situations in which seniors’ health problems are diagnosed only during hospitalization, which in turn may affect long-term hospitalization rates and the length of hospital stay [21,22].

### 1.4. Integration of Key Public Health Theories

To provide a broader understanding of the issues addressed in this study, a literature review was conducted, including several key public health theories. Presenting these theories will allow for a better understanding of the study’s findings. The first is Andersen’s model, which assumes that the use of healthcare services depends on three factors: predisposing factors, enabling factors, and needs. Predisposing factors include characteristics of individuals or communities, such as age, gender, education, and health beliefs. Enabling resources are the conditions that actually enable access to services, such as insurance, access to a physician, infrastructure, and public health programs. Health needs, both subjective and medically assessed, are the direct drivers of healthcare use [23]. In the context of this model, it can be assumed that the intensification of public health programs strengthens both enablers and needs, increasing access to diagnostics and uncovering previously undiagnosed health needs among seniors. As a result, a larger number of programs may be associated with the identification of more complex clinical cases, which, according to the model’s logic, generate higher “real needs” and thus require longer hospitalizations. This means that prolonged hospital stays may not be the result of program ineffectiveness, but rather the consequence of uncovering and treating previously unmet health needs.

Another example of a theory is the socioecological model of health, which assumes that health depends on multilevel factors—individual, social, institutional, environmental, and political [24]. In the context of the obtained results, the observed relationship between a better system structure and improved population health can be interpreted in light of the socioecological model of health: a larger number of programs may increase the detection of unmet needs, improving access to treatment, which may simultaneously result in a short-term increase in hospitalizations or length of stay. The model also emphasizes interactions between levels of care—effective actions require coordination of policies, institutions, and communities, and simultaneous strengthening of all levels can lead to long-term health improvements and reduced inequalities [25].

When analyzing the obtained results, it is also worth considering the social determinants of health, which significantly influence not only health status but also healthcare utilization. Public health literature identifies several key determinants of population health, which often interact: socioeconomic status, geographic location and level of urbanization, access to healthcare, living environment, and institutional and political frameworks [26]. In Poland, research shows significant inequalities in these areas: regions with a higher economic status and better infrastructure have easier access to preventive, primary, and specialist care, which can shorten hospitalizations, while poorer or peripheral regions experience delays in detecting chronic diseases and more frequent, longer hospitalizations [27,28]. The distribution of public health spending and preventive programs also impacts inequality: uneven resource allocation exacerbates differences in access and hospital burden, while effectively planned interventions can improve inequalities in disadvantaged regions. However, the relatively low level of public health spending in Poland limits the effectiveness of such programs [26].

In light of the so-called Law of Inverse Care, which states that the availability of good healthcare is inversely proportional to the health needs of the population [29], it can be assumed that the number of public health programs is not proportional to the level of health needs of seniors. Regions with poorer health indicators have a relatively lower number of preventive measures. At the same time, in areas with more programs in place, there is no decrease in the number of hospitalizations, which may be due to the fact that interventions are more often implemented in more affluent regions characterized by better infrastructure and greater organizational capacity. This mechanism may also explain the observed pattern of longer hospital stays despite the greater number of implemented programs. In regions with higher health needs, where programs are less prevalent, diseases are often detected late and hospitalizations involve more advanced cases requiring longer treatment. In more affluent regions, however, programs may improve detection rates, but they target populations at relatively lower risk, which does not significantly reduce the number of more severe cases requiring hospitalization. The observed results fit the classic pattern of the law of reverse care—where the needs are greatest, the systemic services remain the most modest and the health effects are the most severe.

When analyzing the obtained results, it is also worth considering the complexity of healthcare systems, which are described as adaptive, dynamic, interconnected networks in which a small change in one location can produce a large, unexpected effect elsewhere. Health interventions rarely lead to simple, predictable results, and the effects of actions are often unpredictable and result from the interactions of many system elements, not just from the intervention itself [30]. In this study, increasing the number of healthcare programs in the regions did not lead to shorter hospital stays for seniors, and sometimes even to longer stays. In light of complexity theory, the following could explain these results: each new program changes the behavior of physicians, patients, and institutions, increases the intensity of diagnostics, and identifies more cases of multimorbidity. As a result, patients who require more complex and prolonged treatment are admitted to hospitals. This phenomenon can be described as a “diagnostic cascade,” where greater diagnostic efforts increase disease detection and hospital burdens [31].

Beyond obvious factors such as health status and access to healthcare, social relationships also play a crucial role in achieving health outcomes, as reflected in the social capital theory. This theory posits that differences in health outcomes between regions depend not only on the number of programs or financial resources, but also on the quality of social relationships, trust, and collaborative networks. Communities with high social capital are more likely to engage in health-promoting activities, more effectively overcome barriers to accessing services, and more quickly adopt new programs [32]. The literature distinguishes two types of social capital. Bonding capital encompasses relationships within a group, such as family and neighborhoods, that support participation in programs and facilitate the flow of information. Bridging capital concerns connections between various groups and institutions, such as residents, local government, and healthcare facilities, and is crucial for coordinating activities and reducing systemic barriers [33]. Research from Poland and other countries shows that older participants in health programs are more likely to engage when they have access to strong social support networks, while regions with weaker social capital report lower utilization of available programs [34]. Strong social networks increase the effectiveness of health interventions by disseminating information, encouraging mutual research, providing practical support (e.g., transportation for seniors), and facilitating navigation of the health care system. In regions with weaker social capital, programs often remain “on paper”, which explains why the same number of programs can lead to different health outcomes and do not always translate into shorter hospitalizations or better population health.

## 2. Materials and Methods

### 2.1. Scope of the Study

This study covers the elderly population in Poland, defined as adults aged 65 and over, residing in all 16 voivodeships. Complete data from local and central government units, covering the entire country, were analyzed. The scope of the study was precisely defined both chronologically and substantively. Public health programs and activities targeted at seniors were analyzed in two consecutive years: 2017 and 2018. The information included the number of general activities, preventive activities, support for medical services, promotional and educational activities, training activities, research activities, and other activities. These data reflect the actual intensity of public health policy implementation in individual voivodeships.

In parallel, data were collected on the utilization of healthcare system resources among seniors, focusing on the number of hospitalizations and the average length of hospital stay (average number of days). Data on hospitalizations came from the National Health Fund (NHF) registers and covered the years 2019 and 2020, the period immediately following the analysis of public health program implementation. The database is publicly available to healthcare professionals via an API interface. The statistics come from the national payer database and are published as part of the “open data” project on the Statistics of National Health Fund web page.

The analysis covered the three groups of conditions responsible for the most hospitalizations among older adults: cardiovascular diseases (section E of Polish DRG adaptation), digestive system diseases (section F of Polish DRG adaptation), and musculoskeletal diseases (section H of Polish DRG adaptation). These three groups of diseases were selected because they had the largest shares in the number of hospitalizations among older adults during the study period, and because of their importance in assessing the health needs of the senior population. Including them in the analysis allows for the most reliable determination of potential links between the number of public health tasks and the utilization of hospital care.

Cardiovascular diseases (section E) includes coronary heart disease, heart failure, arrhythmias, conduction disturbances, and other clinically significant cardiac conditions. These diseases have long been a leading cause of morbidity, hospitalization, and mortality in the senior population, making them a key area for assessing the effectiveness of both public health measures and medical care. Digestive diseases (section F) encompass a broad spectrum of chronic and acute conditions among older adults, from inflammatory and infectious diseases to complications of metabolic disorders and cancer. Musculoskeletal diseases (section H) include degenerative joint disease, spine disease, osteoporosis, and other conditions associated with limited functional capacity. In the senior population, these diseases are the dominant cause of chronic ailments, reduced quality of life, and a significant source of hospitalizations—especially in advanced or complicated cases.

The analysis assumed that the impact of public health programs implemented in 2017–2018 on hospitalization rates among seniors may be delayed; therefore, the analysis of hospitalization data was performed for the years 2019–2020. This delay accounts for the time needed for the intervention, potential detection of new cases, referral for treatment, and eventual hospitalization. This approach is consistent with the concept of “lag time to benefit” used in public health literature. This delay should be understood as an analytic assumption aligned with the “lag time to benefit” concept rather than evidence of a precisely defined causal interval at the individual level. In the context of preventive care for older adults, the authors suggest that health benefits, such as reduced disease severity or delayed hospitalizations, may only occur some time after the intervention [35].

At the same time, we emphasize that the assumed two-year lag is a working hypothesis, not empirical confirmation: due to the nature of the data (ecological level of aggregation), it is impossible to precisely estimate the actual time that elapses from intervention to hospitalization for individual patients. Consequently, interpretation of the results should take into account that the observed correlations may reflect both lagged effects and other temporal mechanisms or artifacts resulting from data aggregation.

The scope of this study was intentionally narrowed and excluded a number of areas that, while important from a public health epidemiological perspective, exceed the established analysis objectives. First, the study does not include age groups other than those aged 65 and older. Therefore, the results cannot be directly extrapolated to other demographic groups. Furthermore, the study did not analyze disease categories other than the three groups with the highest hospitalization burden among seniors. Cancer, infectious diseases, mental disorders, respiratory diseases, and endocrine disorders, among others, were not included in the study despite their significant epidemiological role. Their omission is a conscious limitation resulting from the need to maintain a consistent scope of analysis and focus on the most frequently hospitalized diseases.

The study also does not include individual patient health behaviors, such as lifestyle, physical activity, adherence to treatment recommendations, use of outpatient care, or socioeconomic factors. The data used in the analysis are aggregated at the voivodeship level, meaning they do not allow for an assessment of the impact of individual behaviors.

Ultimately, the study does not allow for conclusions about cause-and-effect relationships at the individual level. Analyses based on Pearson correlation coefficients only show trends at the population level, not the direct impact of specific public health interventions on the course of the disease or the treatment decisions of individual patients.

The study should be interpreted with the limitations stemming from its methodology and the adopted scope of analysis in mind. First, the ecological design utilized relies on data aggregated at the voivodeship level, which prevents the assessment of relationships at the individual level and carries the risk of ecological bias. This means that the observed correlations may not reflect the true associations at the level of individual patients. Second, the administrative data sources used may be subject to limitations typical of such databases, such as differences in reporting methods, variability in data quality, and limited detail. Third, the analysis covers only the population aged 65+ and three selected disease groups, which limits the generalizability of the results to other age groups, other diseases, and healthcare systems operating in different organizational settings.

### 2.2. Conceptual Framework

**Figure 1:**
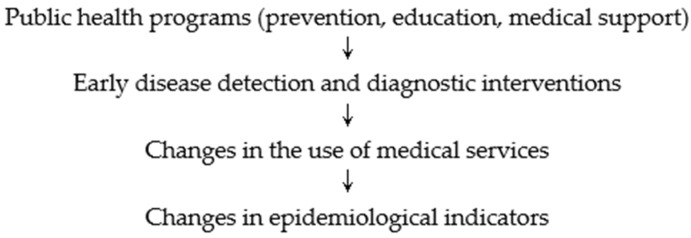
Diagram of logical sequence of events.

The conceptual framework of the study presents a hypothetical path in which the implementation of public health programs targeted at seniors influences health indicators and the utilization of medical services. The assumption is that preventive and educational programs can lead to early disease detection, enabling earlier diagnostic and therapeutic interventions. Early diagnosis results in differentiation of cases in terms of disease severity and duration of hospitalization. Consequently, this may influence hospitalization patterns, reimbursement costs, and observed epidemiological indicators in the senior population. A written outline of the impact of public health interventions on health indicators and the utilization of medical services is presented below (Figure 1).

This diagram presents a logical sequence of events and dependencies that served as the basis for analyzing the correlation between the implementation of public health tasks and health indicators in the studied population of seniors. 

This study did not conduct a sensitivity analysis using alternative lag periods between the implementation of public health measures and observed hospitalization rates. However, considering different scenarios, such as a one- or three-year lag, would be a valuable avenue for future research. Such an approach would allow us to assess whether the observed associations remain stable across lag periods and to more accurately estimate the optimal time period between intervention and health outcome.

The study did not conduct formal robustness checks, such as subgroup analyses or leave-one-out tests. Future work should consider dividing the data by urban and rural areas or high- and low-resource regions to determine whether the observed correlations persist across different sociodemographic and economic contexts.

The diversity of the analyzed public health programs is also a significant limitation. The programs varied in size, cost, and intensity, which could have influenced their effectiveness and made direct comparisons difficult. Future research should develop a classification of programs that takes these characteristics into account and analyze whether individual characteristics modify the relationship between program implementation and hospitalization outcomes in the senior population.

### 2.3. Data Structure and Aggregation

The unit of analysis in this study was the voivodeship, meaning that all collected information, both regarding public health tasks and hospitalization rates, was aggregated across Poland’s sixteen voivodeships. Data from various sources (public health reporting, National Health Fund benefit registers, epidemiological statistics, and demographic data from the Statistics Poland) were combined by voivodeship identifier and processed into indicators standardized to the number of residents aged 65+.

Aggregation at the voivodeship level was conducted in several stages. First, all public health activities reported by local government units and central government units were assigned to voivodeships based on their location. Next, the number of activities in each category and the corresponding financial and population indicators were added together. National Health Fund data were aggregated similarly: the number of hospitalizations within selected DRG sections was summed at the voivodeship level, while median patient lengths of stay were averaged across all services provided in a given region. Epidemiological data on morbidity and mortality were also assigned to voivodeships based on the patients’ region of residence.

The choice of the voivodeship level as the analytical level is justified by the nature of the study and the specificity of the available data. The analysis is ecological in nature; therefore, it refers to relationships at the population level, not individual patients or program participants. Most administrative data is collected in a way that allows for consistent information to be combined at the voivodeship level, ensuring consistency across various data sources. Moreover, voivodeships constitute the unit within which key organizational structures of the healthcare system operate (e.g., voivodeship branches of the National Health Fund, regional health programs), which strengthens the interpretative validity of the correlation analyses conducted.

To enable comparisons between voivodeships with different numbers of seniors, the number of hospitalizations and other indicators related to medical services were standardized to the population aged 65+. Standardization involved converting absolute values to the number of hospitalizations per 1000 people aged 65+. This ensured that the indicators were not distorted by differences in the number of seniors in individual regions.

Standardization procedure based on the example of the Mazowieckie Voivodeship (data for 2018):

X—number of hospitalizations among people aged 65+ in the Masovian Voivodeship in 2018;

Y—number of people aged 65+ in the Masovian Voivodeship in 2018;

Xstd—number of hospitalizations per 1000 people aged 65+;

Xstd = x/y × 1000

Thanks to standardization, hospitalization rates are comparable across voivodeships, regardless of differences in the number of seniors. Other indicators, including reimbursement costs for services and medications and the number of public health activities, were calculated similarly, enabling correlation analysis at an ecological level. Standardization avoided situations in which voivodeships with larger numbers of seniors would artificially inflate the correlations.

### 2.4. Program Classification and Coding

To ensure a consistent analysis of public health interventions, they were classified into seven action categories, consistent with the reporting framework used in monitoring public health tasks. Each task was assigned to a single category based on the description of the purpose, scope of activities, and nature of the intervention indicated by the reporting entity.

General activities encompassed initiatives aimed at broadly supporting the well-being of older adults, including activation, social, and environmental initiatives that did not directly address prevention or diagnosis. These included activities promoting a healthy lifestyle, physical activity, and social participation, as well as the operation of support centers and universities of the third age.

Preventive measures included interventions aimed at disease prevention, early detection, and reducing health risks. This category included vaccination programs (e.g., against influenza or pneumococcal disease), screening tests, and education on the prevention of chronic diseases.

Activities supporting medical services included tasks that complemented or supported medical care, such as rehabilitation, care services, telecare, and specialized services, which ensure the continuation or expansion of patient care.

Educational activities included programs aimed at improving the health literacy of seniors, including information campaigns and workshops on health, chronic diseases, and coping skills in situations requiring healthcare.

Training activities included tasks addressed to medical personnel, caregivers, or those working with seniors, focusing on developing professional competencies and improving the quality of services provided.

Research activities included projects aimed at analyzing the health needs of the population, assessing the effectiveness of interventions, or collecting epidemiological data aimed at improving health policy planning.

Activities identified as “others” included programs that did not fall into any of the above-mentioned categories but were also related to the implementation of local or regional public health activities aimed at seniors.

### 2.5. Identification of Public Health Tasks Targeting Seniors

The identification of public health programs aimed at seniors was based on an analysis of reports submitted by the entities implementing the programs. The primary criterion was the program implementer’s declaration regarding the primary target group–only programs in which the implementers clearly identified people aged 65+ as the primary recipients of their activities, both in the descriptive section and in the report’s classification fields, were included in the analysis. In the public health reporting system, entities can choose from 10 target groups for public health programs: Selected Groups, Seniors, Children and Youth in Total, Primary School Students, Students and Youth in Total, Youth, Total (Youth and Adults), Preschoolers, Young Children, and Pregnant Women. This analysis included programs that targeted seniors. An additional element confirming the targeting of activities at seniors was the selection of the National Health Program operational objective No. 5, “Promoting Healthy and Active Aging”. Programs that identified only general categories as recipients, such as “adults”, “residents of the municipality”, or “people suffering from a specific chronic disease”, were excluded from the analysis, unless they explicitly designated older people as a separate, priority group. This approach meant that some programs could be left out of the analysis even though their impact indirectly affected people aged 65+. However, the lack of a clear senior component in the documentation meant that they could not be considered activities truly targeted at the needs of this group.

### 2.6. Statistical Analysis

Statistical analyses were performed using R Statistics version 4.2.1. Standard criteria for statistical significance were used to interpret the results, with a *p* value of <0.05 considered to indicate a statistically significant correlation. All analyzed indicators were initially standardized to the size of the population of people aged 65+ to account for demographic differences between voivodeships. The results are presented as Pearson correlation coefficients with corresponding *p* values, enabling a clear interpretation of the strength and significance of the relationships.

Range of values r ∈ [−1, 1]:

- r = 1 → full positive correlation (when one variable increases, the other also increases proportionally);
- r = −1 → full negative correlation (when one variable increases, the other decreases);
- r = 0 → no linear relationship between variables.

Interpretation of correlation strength:

- 0.00–0.19 → very weak;
- 0.20–0.39 → weak;
- 0.40–0.59 → moderate;
- 0.60–0.79 → strong;
- 0.80–1.00 → very strong.

### 2.7. Summary of the Analytical Sequence

The study adopted the following analytical sequence: first, demographic and epidemiological data, data on hospitalizations, and public health tasks implemented for the population of people aged 65+ were obtained and prepared. Next, public health programs were classified and coded, tasks targeted at seniors were identified, and data were aggregated at the administrative unit level (voivodeship). The next step was to standardize the indicators based on the size of the senior population and verify the assumptions necessary for Pearson correlation analysis. Finally, correlation coefficients were calculated, and the results were assessed for statistical significance.

## 3. Results

Table 1, Table 2 and Table 3 present Pearson’s r correlation coefficients between the public health tasks carried out for seniors and the incidence and duration of hospitalizations for three groups of diseases that, according to our analyses, were the primary causes of hospitalizations. Positive correlations were observed between hospitalization time and the 3 disease groups examined.

The analysis revealed statistically significant, positive correlations between the intensity of implementation of programs and activities targeted at seniors in 2017 and the duration of hospitalization due to diseases from sections E, F, and H in 2019. The strongest relationships were observed for digestive system diseases, particularly in relation to the total number of programs and programs supporting medical services. In all disease groups analyzed, positive correlations were observed for both the total number of programs and activities supporting medical services, as well as the “other” category (Table 1).

In 2020, significant positive correlations were observed between the number of programs and activities targeted at seniors in 2017 and the duration of hospitalizations for heart, digestive, and musculoskeletal conditions. The highest correlation coefficients were observed for programs supporting medical services and the total number of programs for digestive and musculoskeletal conditions (Table 2).

The analysis revealed significant positive correlations between the number of programs and activities targeted at seniors and the duration of hospitalization for musculoskeletal disorders and heart and digestive system diseases. The strongest correlation was observed for programs supporting medical services for musculoskeletal and heart diseases (Table 3).

## 4. Discussion

The analyses presented indicate a positive correlation between the number of public health tasks—particularly those supporting medical services and those categorized as ‘other’—and prolonged hospital stays among seniors with three specific groups of diseases: cardiovascular, digestive, and musculoskeletal. While many studies have found that public health interventions tend to either reduce or show no significant increase in hospitalization duration, this analysis suggests otherwise. For instance, a review by Siddique SM [36] found that numerous interventions were associated with shorter hospital stays. Likewise, a meta-analysis by Hun-O’Connor [37] found that effective discharge planning typically reduces length of stay, with a mean difference of about ≈0.71 days among older adults. In contrast, a long-term cohort study conducted over 20 years in Italy revealed that, despite shorter hospitalization times, the readmission rate within 30 days post-discharge increased. The findings suggest that shorter stays may be associated with lower-quality discharges or a shift in responsibility toward community care. In the authors’ opinion, several factors may explain the study’s results.

### 4.1. Program Effect on Diagnostic Intensity

Implementing a larger number of comprehensive public health programs naturally leads to an increase in screening. As a result, previously undiagnosed health problems are more frequently detected. This, in turn, generates more diagnostic tests, consultations, confirmatory tests, and consequently, the need for hospitalization. Thus, through the so-called “diagnostic cascade”, greater program intensity not only increases the frequency of disease detection but can also lead to longer hospital stays for patients who require more complex treatment. Such processes are well documented in the literature. In the case of infectious diseases, engaging multiple healthcare providers in screening projects increased the number of patients, some of whom were further tested and ultimately diagnosed with the disease—this occurred less frequently, but at a stage requiring intensive care [38]. Similarly, in oncology, population-based screening programs (e.g., mammography, colonoscopy) lead to the detection of preclinical changes, which may require further diagnosis, biopsy, treatment, and sometimes hospitalization [39]. With the development of methods (e.g., biochemical, molecular, or genetic) and the expansion of diagnostic resources, the number of complex cases is also increasing, which translates into a greater demand for hospitalizations and treatment [40].

Consequently, even if the program is preventative in nature and aims to identify diseases early, a “side effect” will be increased diagnostic and therapeutic intensity, which may lead to increased length of hospital stays. Therefore, when assessing the impact of the program, it should be considered that some of the increase in hospitalizations or treatment duration may result not so much from poor quality of care, but from the effective diagnosis of previously undiagnosed conditions.

### 4.2. Case Mix and Multimorbidity

Multimorbidity may be a key factor explaining the observed increase in length of hospital stays under the program. There is ample evidence in the literature that patients with multiple chronic conditions are more likely to require hospitalization, experience complications more frequently, and have longer hospital stays. For example, a large, international retrospective study of over 126,000 patients with multimorbidity demonstrated that specific combinations of conditions were strongly correlated with prolonged hospitalization—for the most complex sets of conditions, the median length of stay was up to 13 days longer [41]. Similarly, in a Swiss administrative study covering over 2.2 million hospitalizations, patients with multiple chronic conditions spent an average of approximately 2.7 more days in the hospital than those without comorbidities [42]. In the context of chronic diseases such as chronic obstructive pulmonary disease (COPD), comorbidities have been strongly associated with longer hospitalization durations [43]. In light of this evidence, it is possible that the program, particularly if focused on high-risk populations or groups with chronic conditions, preferentially reaches patients with more severe and complex health problems. Consequently, an increase in hospitalizations or an increase in average length of stay may not reflect a negative impact of the program but rather the program’s effectiveness in identifying and attracting individuals requiring intensive care, and such individuals naturally have greater treatment needs and longer lengths of stay.

### 4.3. Health System Context and Outpatient Care

An important element that can strongly modulate program-related outcomes is the structure and accessibility of outpatient and community care. Numerous studies indicate that robust, well-organized outpatient and community care can reduce hospitalization rates or shorten hospital stays, which in turn influences how we interpret observed changes in aggregated data. For example, one retrospective analysis found that greater distance from the nearest clinic (i.e., poorer outpatient access) was correlated with a higher risk of hospitalization for ambulatory-sensitive conditions, particularly in older adults [44]. Similarly, another study showed that for patients with chronic conditions, when a 24-h/easy-access outpatient care model was introduced, both the frequency of admissions and the average length of hospital stay decreased [45]. On the other hand, if outpatient or community care is underdeveloped or difficult to access in a given region, patients with health problems that could receive consultations and treatment in an outpatient setting may be hospitalized more often. In this context, regional differences in the availability of outpatient care may obscure the true impact of the program as some hospitalizations or longer stays may be the result of deficiencies in the primary/outpatient care system rather than a direct consequence of the program itself. Therefore, when interpreting data on the program’s impact on hospitalizations and length of stay, it is important to consider differences in the availability and quality of outpatient care between regions.

### 4.4. Program Quality and Design

Program heterogeneity, both in terms of its intensity, quality, and degree of integration with clinical care, can significantly impact observed outcomes. Not all programs are equally effective; some may offer a comprehensive, well-coordinated package of preventive, educational, and diagnostic activities while others operate on a limited scale or are less well-organized. These differences may indirectly shape hospital patterns: programs better integrated with outpatient and inpatient care may lead to earlier detection of conditions, increasing the number of diagnostic tests and sometimes extending hospital stays, while less intensive or fragmented programs may not produce such effects. Therefore, interpretation of results requires consideration that observed changes in length of stay or the number of hospitalizations may be partially the result of differences in the quality and structure of the programs themselves, not simply their number.

### 4.5. International Perspective

The results of this analysis may be particularly useful for countries with healthcare systems similar to Poland’s, especially those characterized by similar levels of public health funding, outpatient care structures, and demographic challenges. Countries with comparable organizational models could use the presented observations as a benchmark for evaluating their own prevention programs. At the same time, these results point to the need for cross-country comparative studies to better understand whether similar mechanisms—such as diagnostic intensity or the impact of social inequalities—also exist in other systems. Such international analyses could provide more general conclusions and support the development of more effective public health policies.

### 4.6. Limitations

The correlation data presented does not allow for establishing causality. It remains unclear whether the programmes cause more extended hospital stays or whether more programmes are implemented in areas with longer stays. Thus, further, more in-depth research is required.

It’s worth emphasizing that the analysis is based on data aggregated at the regional or city level, meaning that the presented results reflect relationships occurring at the population level, not individual patients. Interpretation of the effects is therefore limited by the ecological nature of the data: observed relationships may not necessarily exist at the individual level, and their extrapolation to individuals could lead to ecological bias. Ecological data show relationships at the level of groups, such as hospitals or regions. If hospitals with a larger program presence have a longer average length of stay, this does not automatically mean that a specific patient enrolled in the program had a longer stay.

Despite these limitations, the ecological approach remains appropriate in this study because public health programs are planned, financed, and evaluated at the level of entire populations, regions, or institutions, not individual patients. Policy decisions regarding resource allocation, the introduction of new programs, and the assessment of their effectiveness are made at this aggregated scale. Therefore, analyzing data at the group level allows us to answer questions relevant to decision-makers and effectively supports the planning and evaluation of health policies, even if it does not allow for inferences about the effects on individual individuals.

In the analysis of ecological data, the possibility of confounding factors must be considered. Potential variables that may significantly influence the observed results include the socioeconomic status of the population, regional income levels, hospital capacity, the density of available outpatient services, and the baseline health status of the population. These factors can modify both participation in health programs and the average length of stay or other measured indicators, making it difficult to clearly interpret the associations observed at the aggregate level. Because these confounding factors were not accounted for in the analysis, the observed correlations may in part reflect underlying regional differences—such as socioeconomic status, hospital resources, or the baseline health status of the population—rather than solely the scale of the program.

The correlation analysis used in this study also did not explicitly control for the influence of population aging as a potential confounding factor. Population aging is strongly associated with increased hospitalization rates, multimorbidity, and increased clinical complexity, as confirmed by studies of hospitalization trends in older age groups [46]. Therefore, it is difficult to clearly distinguish the impact of public health programs on hospitalization rates from demographic changes without using appropriate techniques such as age standardization or multivariate modeling with control for the age structure of the population, which are commonly used in epidemiological studies to eliminate the age effect as a confounding factor [47]. Future studies should include age-disaggregated data or use appropriate statistical techniques to better assess the independent impact of public health interventions on the frequency and duration of hospitalizations among seniors, regardless of demographic trends in population aging.

Future research should consider an approach that accounts for both confounding factors and regional variation. One approach is to use multivariate regression models that, in addition to a key variable such as program participation, also include sociodemographic and resource-related variables such as the population’s socioeconomic status, regional income, hospital capacity, and the availability of outpatient services. Such models allow for the assessment of the program’s impact while controlling for potential confounding factors.

Additionally, the use of multilevel (hierarchical) models is recommended, which enable simultaneous analysis of data at various levels: program intensity, hospital outcomes, and regional characteristics. This approach allows for accounting for differences between hospitals and regions, as well as for the relationship between program levels and patient outcomes, providing a more comprehensive picture of the effects of public health interventions.

The replicability of this study is limited by incomplete details of some methodological procedures and the need to rely on administrative data, which may not contain all relevant information. Furthermore, changes in the way data are reported or classified in the future may make direct replication of the analysis difficult. Therefore, the results should be interpreted with these limitations in mind and with caution when generalizing to other contexts.

Confounding factors were not included in this analysis, primarily due to limitations in the available data and the exploratory nature of the work. The analysis primarily aimed to identify general patterns and generate hypotheses for further, more detailed research that controls for sociodemographic and resource variables.

It should be emphasized that the use of correlations in an ecological study is an appropriate first step in exploring the relationship between implemented programs and hospitalization rates but it does not allow for establishing program effectiveness or causality. This analysis is exploratory in nature and generates hypotheses that can be tested in future studies with more advanced methodological design.

The main strengths of the methodological approach include the use of nationwide administrative data, standardization of indicators for the senior population, and systematic classification of public health programs. Weaknesses include the limitations of the ecological design, the lack of control over individual variables, and the possible inaccuracies of administrative data. This study should be considered a starting point for more complex, controlled analyses that could better determine causality and the mechanisms of action of public health programs.

## 5. Conclusions

The aim of this study was to examine the relationship between public health programs targeted at seniors and hospitalization rates in the population aged 65 and older in Poland. The analysis revealed a positive correlation between the number of public health programs implemented and prolonged hospitalizations among seniors, which may be due to more intensive diagnostics, the discovery of previously undiagnosed conditions, and a greater proportion of patients with multimorbidity. The results suggest that these programs more often identify individuals with more complex needs, and regional differences in the availability of outpatient care and system resources may further modify the observed effects. Consequently, the number of programs does not automatically translate into shorter hospital stays, and these relationships require further analysis, taking into account confounding factors and the quality of interventions.

Based on the obtained results, several recommendations can be formulated for decision-makers and healthcare system staff:

1. The number and availability of public health interventions for seniors should be increased.
2. Rehabilitation and care programs that integrate with medical services should be supported to improve continuity of care.
3. The effectiveness of regional public health programs should be continuously monitored and assessed to identify best practices.
4. Outpatient and community care should be strengthened to reduce the need for hospitalization for typically outpatient cases.
5. Collaboration between central and local government units should be facilitated in planning and financing programs for seniors.
6. Future initiatives should take into account differences between urban and rural areas and between regions with different levels of resources.
7. The transparency and detail of administrative data should be increased to enable future analyses and improve the generalizability of results.

## Figures and Tables

**Table 1:** Correlation coefficients between various programmes and activities geared towards seniors in 2017, along with the number and duration of hospitalizations due to diseases from sections E, F, and H in 2019.

Disease Section	Type of Programme/Activity	Correlation Coefficient (r)
Heart disease (E)	Total number of programmes	0.501 [0.007, 0.798] *
	Programmes supporting medical services ^a^	0.574 [0.109, 0.833] *
	“Other” activities ^b^	0.519 [0.031, 0.810] *
Diseases of the digestive system (F)	Total number of programmes	0.610 [0.164, 0.849] *
	“General” programmes ^c^	0.526 [0.041, 0.810] *
	Prevention programmes ^d^	0.498 [0.003, 0.797] *
	Programmes supporting medical services	0.601 [0.150, 0.845] *
	“Other” activities	0.514 [0.025, 0.805] *
Diseases of the musculoskeletal system (H)	Total number of programmes	0.582 [0.121, 0.836] *
	“General” programmes	0.498 [0.003, 0.797] *
	Programmes supporting medical services	0.596 [0.142, 0.843] *
	“Other” activities	0.558 [0.086, 0.825] *

* *p* < 0.05. a—Programmes supporting medical services included tasks that complemented or supported medical care; b—Activities identified as “others” included programs that did not fall into any of the above-mentioned categories but were also related to the implementation of local or regional public health activities aimed at seniors; c—General activities encompassed initiatives aimed at broadly supporting the well-being of older adults, including activation, social, and environmental initiatives that did not directly address prevention or diagnosis; d—Preventive measures included interventions aimed at disease prevention, early detection, and reducing health risks.

**Table 2:** Correlation coefficients between the programmes and activities implemented for seniors in 2017 and the number and duration of hospitalizations due to diseases from sections E, F, and H in 2020.

Disease Section	Type of Programme/Activity	Correlation Coefficient (r)
Heart disease (E)	Programmes supporting medical services ^a^	0.599 [0.147, 0.844] *
	“Other” activities ^b^	0.581 [0.112, 0.836] *
Diseases of the digestive system (F)	Total number of programmes	0.623 [0.184, 0.855] **
	“General” programmes ^c^	0.544 [0.066, 0.819] *
	Programmes supporting medical services ^d^	0.599 [0.147, 0.844] *
	“Other” activities	0.498 [0.003, 0.797] *
Diseases of the musculoskeletal system (H)	Total number of activities	0.555 [0.082, 0.824] *
	Programmes supporting medical services	0.629 [0.194, 0.857] **
	“Other” activities	0.517 [0.029, 0.806] *

* *p* < 0.05; ** *p* < 0.001. a—Programmes supporting medical services included tasks that complemented or supported medical care; b—Activities identified as “others” included programs that did not fall into any of the above-mentioned categories but were also related to the implementation of local or regional public health activities aimed at seniors; c—General activities encompassed initiatives aimed at broadly supporting the well-being of older adults, including activation, social, and environmental initiatives that did not directly address prevention or diagnosis; d—Preventive measures included interventions aimed at disease prevention, early detection, and reducing health risks.

**Table 3:** Correlation coefficients between senior programs implemented in 2018 and hospitalization frequency and duration for diseases in sections E, F, and H in 2020.

Disease Section	Type of Programme/Activity	Correlation Coefficient (r)
Heart disease (E)	Programmes supporting medical services ^a^	0.650 [0.228, 0.867] **
Diseases of the digestive system (F)	Total number of programmes	0.562 [0.092, 0.827] *
	Programmes supporting medical services	0.587 [0.129, 0.839] *
Diseases of the musculoskeletal system (H)	Programmes supporting medical services	0.700 [0.313, 0.888] **

* *p* < 0.05; ** *p* < 0.001. a—Programmes supporting medical services included tasks that complemented or supported medical care.

## Data Availability

All the data is presented in the article.

## References

[1] World Health Organization: Ageing and Health. https://www.who.int/news-room/fact-sheets/detail/ageing-and-health.

[2] OECD Elderly Population. https://www.oecd.org/en/data/indicators/elderly-population.html.

[3] Minister for Senior Policy, Information on the Situation of Elderly People in Poland for 2024, Warsaw 2025. https://www.gov.pl/attachment/84772355-b55e-49e1-af90-817518e42503.

[4] (2023). State of Health in the EU. Series: State of Health in the EU.

[5] Statistics Poland, The Situation of Elderly People in Poland in 2023, Warsaw, Białystok 2024. https://stat.gov.pl/files/gfx/portalinformacyjny/pl/defaultaktualnosci/6002/2/6/1/sytuacja_osob_starszych_w_polsce_w_2023_r.pdf.

[6] Pobrotyn P., Susło R., Witczak I.T., Rypicz Ł., Drobnik J. (2020). An analysis of the costs of treating aged patients in a large clinical hospital in Poland under the pressure of recent demographic trends. Arch. Med. Sci..

[7] Po H.W., Lin F.J., Cheng H.J., Huang M.L., Chen C.Y., Hwang J.J., Chiu Y.W. (2023). Factors Affecting the Effectiveness of Discharge Planning Implementation: A Case-Control Cohort Study. J. Nurs. Res..

[8] Bayer-Oglesby L., Zumbrunn A., Bachmann N., on behalf of the SIHOS Team (2022). Social inequalities, length of hospital stay for chronic conditions and the mediating role of comorbidity and discharge destination: A multilevel analysis of hospital administrative data linked to the population census in Switzerland. PLoS ONE.

[9] Umegaki H. (2024). Hospital-associated complications in frail older adults. Nagoya J. Med. Sci..

[10] Mackel E.M., Breda K.L. (2024). Living well: Evidence-based self-management of chronic diseases for community-dwelling older adults. Nursing.

[11] Zhu J., Wang W., Wang J., Zhu L. (2022). Change in coronary heart disease hospitalization after chronic disease management: A programme policy in China. Health Policy Plan..

[12] OECD (2025). The Economic Benefit of Promoting Healthy Ageing and Community Care.

[13] Wu J., Chen D., Li C., Wang Y. (2024). Effect of community-based public health service on health-related quality of life among middle-aged and older adults with chronic diseases in China. BMC Public Health.

[14] Chandrashekhar A., Thakur H.P. (2022). Efficacy of Government-Sponsored Community Health Programs for Older Adults: A Systematic Review of Published Evaluation Studies. Public Health Rev..

[15] Chica-Pérez A., Dobarrio-Sanz I., Ruiz-Fernández M.D., Correa-Casado M., Fernández-Medina I.M., Hernández-Padilla J.M. (2023). Effects of home visiting programmes on community-dwelling older adults with chronic multimorbidity: A scoping review. BMC Nurs..

[16] OECD (2025). Health at a Glance.

[17] Yu Y., Zhang J., Petrovic M., Zhang X., Zhang W.H. (2024). Utilization of home- and community-based services among older adults worldwide: A systematic review and meta-analysis. Int. J. Nurs. Stud..

[18] Balqis-Ali N.Z., Jawahir S., Chan Y.M., Lim A.W.Y., Azlan U.W., Shaffie S.S.M., Fun W.H., Lee S.W.H. (2024). The impact of long-term care interventions on healthcare utilisation among older persons: A scoping review of reviews. BMC Geriatr..

[19] Sowada C., Sagan A., Kowalska-Bobko I. (2022). Poland: Health System Summary.

[20] Michalska K., Domagała A. (2025). Addressing the health workforce crisis in Poland from the key stakeholders’ perspectives–a qualitative study. BMC Health Serv. Res..

[21] Rzecznik Praw Pacjenta, Sprawozdanie Rzecznika Praw Pacjenta z Przestrzegania Praw Pacjenta w Roku 2024, Warszawa 2025. https://www.gov.pl/web/rpp/sprawozdanie-za-2024-rok.

[22] Barometr WHC, Polacy w Kolejkach, Raport Dotyczący Zmian w Dostępie do Gwarantowanych Świadczeń Zdrowotnych w Polsce, nr 01/11/2022. https://www.gov.pl/attachment/bb888f27-bc5f-478c-9def-bb1703c682d8.

[23] Ydstebø A.E. (2020). Home-Dwelling Persons with Dementia. The Impact of Individual and Organizational Factors on the Use of Health Resources and Quality of Life. Ph.D. Thesis.

[24] Korom B., Malloy M., Remmers C., Cevilla M., Dione K., Papanek P., Condit J., Nelson D. (2023). “It’s about being healthy”; a novel approach to the socio-ecological model using family perspectives within the Latinx community. BMC Public Health.

[25] Mesa-Vieira C., Gonzalez-Jaramillo N., Díaz-Ríos C., Pano O., Meyer S., Menassa M., Minder B., Lin V., Franco O.H., Frahsa A. (2023). Urban Governance, Multisectoral Action, and Civic Engagement for Population Health, Wellbeing, and Equity in Urban Settings: A Systematic Review. Int. J. Public Health.

[26] Wojtyniak B., Goryński P., Moskalewicz B. (2013). The health situation of the Polish population and its determinants. Nowotw. J. Oncol..

[27] Zienkiewicz T., Zalewska A., Zienkiewicz E. (2025). Regional Disparities and Determinants of Paediatric Healthcare Accessibility in Poland: A Multi-Level Assessment of Socio-Economic Drivers and Spatial Convergence (2010–2023). Sustainability.

[28] Zienkiewicz T., Klatka M., Zienkiewicz E., Klatka J. (2022). Determinants and disparities in access to paediatricians in Poland. BMC Prim. Care.

[29] Mercer S.W., Lunan C.J., MacRae C., Henderson D.A., Fitzpatrick B., Gillies J., Guthrie B., Reilly J. (2023). Half a century of the inverse care law: A comparison of general practitioner job satisfaction and patient satisfaction in deprived and affluent areas of Scotland. Scott. Med. J..

[30] Carroll Á., Collins C., McKenzie J., Stokes D., Darley A. (2023). Application of complexity theory in health and social care research: A scoping review. BMJ Open.

[31] McGill E., Er V., Penney T., Egan M., White M., Meier P., Whitehead M., Lock K., de Cuevas R.A., Smith R. (2021). Evaluation of public health interventions from a complex systems perspective: A research methods review. Soc. Sci. Med..

[32] Zawisza K., Sekuła P., Gajdzica M., Tobiasz-Adamczyk B. (2024). Social capital and all-cause mortality before and during the COVID-19 pandemic among middle-aged and older people: Prospective cohort study in Poland. Soc. Sci. Med..

[33] Degli Antoni G., Grimalda G. (2024). Is social capital bridging or bonding? Evidence from a field experiment with association members. Theory Decis..

[34] Cianciara D., Lewtak K., Poznańska A., Piotrowicz M., Gajewska M., Urban E., Sugay L., Rutyna A. (2023). Participation in Population Health Interventions by Older Adults in Poland: Barriers and Enablers. Int. J. Environ. Res. Public Health.

[35] Holmes H.M., Min L., Boyd C. (2014). Lag Time to Benefit for Preventive Therapies. JAMA.

[36] Siddique S.M., Tipton K., Leas B., Greysen S.R., Mull N.K., Lane-Fall M., McShea K., Tsou A.Y. (2021). Interventions to Reduce Hospital Length of Stay in High-risk Populations: A Systematic Review. JAMA Netw. Open.

[37] Hunt-O’Connor C., Moore Z., Patton D., Nugent L., Avsar P., O’Connor T. (2021). The effect of discharge planning on length of stay and readmission rates of older adults in acute hospitals: A systematic review and meta-analysis of systematic reviews. J. Nurs. Manag..

[38] Rahman T., Wells W.A., Ramis O., Kamineni V.V., Bakker M.I., Matiku S., Brouwer M., Creswell J. (2025). Engaging private providers to enhance tuberculosis detection and notification: Evidence from TB REACH-Supported projects. BMC Public Health.

[39] Profibaza, Badania Przesiewowe w Kierunku Nowotworów. https://profibaza.pzh.gov.pl/Opieka_zdrowotna/Badania%20przesiewowe%20w%20kierunku%20nowotwor%C3%B3w.

[40] Liu Q., Jin X., Cheng J., Zhou H., Zhang Y., Dai Y. (2023). Advances in the application of molecular diagnostic techniques for the detection of infectious disease pathogens (Review). Mol. Med. Rep..

[41] Aubert C.E., Schnipper J.L., Fankhauser N., Marques-Vidal P., Stirnemann J., Auerbach A.D., Zimlichman E., Kripalani S., Vasilevskis E.E., Robinson E. (2020). Association of patterns of multimorbidity with length of stay: A multinational observational study. Medicine.

[42] Mueller M., Huembelin M., Baechli C., Wagner U., Schuetz P., Mueller B., Kutz A. (2021). Association of in-hospital multimorbidity with healthcare outcomes in Swiss medical inpatients. Swiss. Med. Wkly..

[43] Jankowski M., Bochenek B., Wieczorek J., Figurski M., Gruszczyńska M., Goryński P., Pinkas J. (2023). Epidemiological Characteristics of 101,471 Patients Hospitalized with Chronic Obstructive Pulmonary Disease (COPD) in Poland in 2019: Multimorbidity, Duration of Hospitalization, In-Hospital Mortality. Adv. Respir. Med..

[44] Iba A., Tomio J., Sugiyama T., Abe K., Yamada I., Kobayashi Y. (2023). Association between spatial access and hospitalization for ambulatory care sensitive conditions: A retrospective cohort study using claims data. SSM-Popul. Health.

[45] Schlünsen A.D.M., Christiansen D.H., Fredberg U., Vedsted P. (2022). Effectiveness of a 24-hour access outpatient clinic for patients with chronic conditions in hospital outpatient follow-up: A registry-based controlled cohort study of healthcare utilisation and mortality. Integr. Health J..

[46] Piñeiro-Fernández J.C., Rabuñal-Rey R., Romay-Lema E., Chantres-Legaspi Y., Santos-Martínez A.M., Besteiro-Balado Y., Suárez-Gil R., Pértega-Díaz S. (2025). Trends in hospital admissions and clinical complexity in centenarians: A nationwide population-based study in Spain (2004–2020). Eur. Geriatr. Med..

[47] Faitna P., Bottle A., Klaber B., Aylin P.P. (2024). Has multimorbidity and frailty in adult hospital admissions changed over the last 15 years? A retrospective study of 107 million admissions in England. BMC Med..

